# Highly Enhanced OER Performance by Er-Doped Fe-MOF Nanoarray at Large Current Densities

**DOI:** 10.3390/nano11071847

**Published:** 2021-07-16

**Authors:** Yan Ma, Yujie Miao, Guomei Mu, Dunmin Lin, Chenggang Xu, Wen Zeng, Fengyu Xie

**Affiliations:** 1 College of Chemistry and Materials Science, Sichuan Normal University, No. 5, Jing’an Road, Chengdu 610068, China; 20191201038@stu.sicnu.edu.cn (Y.M.); yujie@stu.sicnu.edu.cn (Y.M.); guomei@stu.sicnu.edu.cn (G.M.); ddmd222@sicnu.edu.cn (D.L.); chenggangxu@163.com (C.X.); 2 School of Chemistry and Chemical Engineering, Chongqing University, Shapingba District, Chongqing 401331, China; wenzeng@cqu.edu.cn

**Keywords:** rare-earth doping, MOFs, large current, electronic structure

## Abstract

Great expectations have been held for the electrochemical splitting of water for producing hydrogen as a significant carbon-neutral technology aimed at solving the global energy crisis and greenhouse gas issues. However, the oxygen evolution reaction (OER) process must be energetically catalyzed over a long period at high output, leading to challenges for efficient and stable processing of electrodes for practical purposes. Here, we first prepared Fe-MOF nanosheet arrays on nickel foam via rare-earth erbium doping (Er_0.4_ Fe-MOF/NF) and applied them as OER electrocatalysts. The Er_0.4_ Fe-MOF/NF exhibited wonderful OER performance and could yield a 100 mA cm^−2^ current density at an overpotential of 248 mV with outstanding long-term electrochemical durability for at least 100 h. At large current densities of 500 and 1000 mA cm^−2^, overpotentials of only 297 mV and 326 mV were achieved, respectively, revealing its potential in industrial applications. The enhancement was attributed to the synergistic effects of the Fe and Er sites, with Er playing a supporting role in the engineering of the electronic states of the Fe sites to endow them with enhanced OER activity. Such a strategy of engineering the OER activity of Fe-MOF via rare-earth ion doping paves a new avenue to design other MOF catalysts for industrial OER applications.

## 1. Introduction

As a form of clean energy, hydrogen energy is of crucial importance in addressing the global energy crisis and greenhouse gas issues caused by increased consumption of fossil fuels sources. According to literature, water electrolysis technology is an effective method of obtaining high purity hydrogen and includes two half reactions: the cathodic hydrogen evolution reaction (HER) and anodic oxygen evolution reaction (OER). Compared with HER, the OER suffers from sluggish kinetics and thus requires higher energy input to overcome the reaction energy barrier [1,2,3]. Although RuO_2_ and IrO_2_ have been verified to be the most active OER catalysts, the high cost and scarcity of such catalysts restrict their large-scale application [4,5]. Thus, it is critical to find non-noble metals that have high activity, low cost, and are more stable at large currents for applications.

A great deal of research has been performed in order to investigate highly efficient non-noble metal-based OER catalysts, including metal oxides [6], chalcogenides [7,8], hydroxides [9], phosphides [10], nitrides [11], and metal organic frameworks (MOFs) [12,13,14]. Among them, MOFs exhibit superior OER activity. However, most reported MOF electrocatalysts have been evaluated in accordance with overpotentials and long-term stability under low current density, mostly less than 100 mA cm^−2^, and these assessment criteria are not realistic for industrial applications [15]. To be used for industrial applications, OER catalysts must deliver large-current-density (500 mA cm^−2^) OER at low overpotentials. Accordingly, it is highly attractive to design and develop efficient OER electrocatalysts based on earth-abundant MOF alternatives at large current densities. Recent works have demonstrated modifying of the electronic structure of active sites by introducing foreign atoms as an effective strategy to enhance electrocatalysts’ intrinsic activity. Specifically, Gao et al. systematically designed transition-metal-doped NiFe LDHs (using Co, Mn, and Cr metals). Combined with theory analysis, they found that the enhanced intrinsic OER activity was due to modifying the electronic structure of the intermediate site after Co, Mn, and Cr doping [16]. Ma et al. proved that Cu-doped Ni_3_S_2_/Co_3_S_4_ could not only increased the specific surface area but also alter the electron density of Ni/Co sites [17]. For the past few years, rare-earth elements have received more and more attention on account of the 4f orbitals partial filling [18,19]. Importantly, the doping of rare-earth elements can effectively regulate the electronic and geometric structures of electrocatalysts, thus improving their electrical conductivity and internal catalytic activity. For instance, Wan et al. synthesized La-doped In(OH)_3_, improving the visible light absorption capability for the photo-reduction of carbon dioxide [20]. Xu et al. reported that Ni–Fe–Ce–LDH microcapsules exhibited a high OER activity owing to the optimized electronic structure of 3D LDHs through Ce species doping [21]. Ahmad et al. found that doped Eu and Tb ions could enter the lattice of ZnO, and the resulting ZnO had a large specific surface area and high porosity [22]. However, there still are few reports about enhancing the intrinsic OER activity of MOFs by rare-earth element doping, especially under large currents [23,24].

**Scheme 1:**
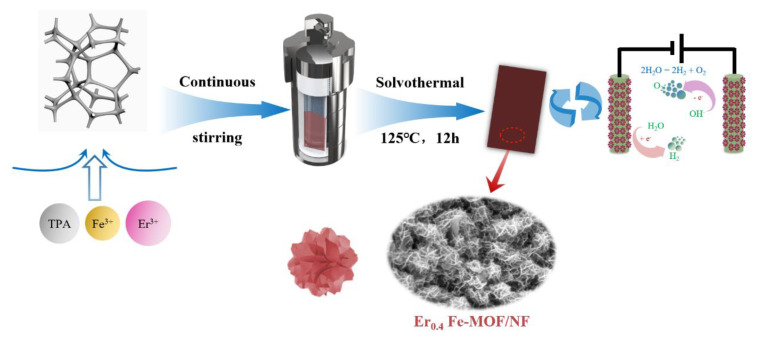
Schematic illustrating the fabrication process for Er_0.4_ Fe-MOF/NF.

To tackle the above challenge, we rationally designed and synthesized Fe-MOF nanosheet arrays grown on 3D nickel foam (NF) via Er doping (Er_0.4_ Fe-MOF/NF) as an advanced OER catalyst (Scheme 1). Comparing with the precursor Fe-MOF/NF, the achieved Er_0.4_ Fe-MOF/NF exhibited an ultralow overpotential of 248 mV to drive 100 mA cm^−2^ current density and showed an outstanding long-term electrochemical durability of more than 100 h without obvious decay. Moreover, the overpotentials of 297 mV and 326 mV were needed to drive 500 and 1000 mA cm^−2^, indicating its promising application. This work provides some inspiration for reasonably designing efficient and stable MOFs for industrial applications.

## 2. Materials and Methods

All chemical regents used in this work were analytically pure. Nickel foam (NF, area: 2 cm × 3 cm) was bought from Shenzhen Green and Creative Environmental Science and Technology Co., Ltd (Shenzhen, China). Ferric nitrate nonahydrate (Fe(NO_3_)_3_·9H_2_O), erbium (III) nitrate hexahydrate (Er(NO_3_)_3_·6H_2_O; Aladdin), terephthalic acid (C_8_H_6_O_4_, TPA; Aladdin), hydrochloric acid (HCl; Aladdin), N, N-dimethylformamide (DMF; Beijing Chemical Industry Group Co., Ltd, Beijing China), ethanol, and deionized water were used.

### 2.1. Preparation of Er_0.4_ Fe-MOF on NF

Er_0.4_ Fe-MOF/NF was prepared via a one-step solvothermal method. First, 1 mmol Fe(NO_3_)_3_·9H_2_O (0.4040 g), 0.4 mmol Er(NO_3_)_3_·6H_2_O (0.1845 g), and 3 mmol TPA (0.4980 g) were added to 35 mL of DMF while stirring uniformly at room temperature. Subsequently, 2.5 mL of ethanol and 2.5 mL of deionized water were added. This mixture was stirred vigorously for half an hour and rapidly transferred into a 50 mL autoclave. The treated NF (2 × 3 cm) was then placed in the homogeneous solution. The solution was put in one Teflon-lined stainless autoclave and reacted at 125 °C for 12 h. Finally, it was naturally cooled down to room temperature, the material was washed several times with deionized water, and it was dried in a vacuum oven at 60 °C. The mass loading of Er_0.4_ Fe-MOF/NF was about 1.18 mg cm^−2^.

### 2.2. Preparation of Fe-MOF on NF

The Fe-MOF/NF was synthesized using the above-mentioned process without Er(NO_3_)_3_·6H_2_O doping.

### 2.3. Preparation of RuO_2_ on NF

RuO_2_/NF was prepared in accordance with the reported work. First, 2.61 g of RuCl_3_·3H_2_O and 1.0 mL KOH (1.0 M) were added into 100 mL of distilled water and stirred for 45 min at a temperature of 100 °C. The precipitate was collected via centrifugation, washed several times with distilled water, and then dried in a vacuum oven at 60 °C. Finally, the product was annealed at 300 °C for 3 h in an air atmosphere to obtain RuO_2_ powder. Catalyst ink was prepared by dispersing 22.5 mg of catalyst into 490 µL of water/ethanol (volume ratio, *v*/*v* = 1:1) and 20 µL of 5 wt% Nafion using sonication treatment for 30 min. Subsequently, 13.0 µL of the RuO_2_ ink (containing 0.295 mg of RuO_2_) was loaded onto a bare NF of 0.25 cm^−2^ in geometric area (mass loading: 1.18 mg cm^−2^).

### 2.4. Electrochemical Measurements

All electrochemical tests were measured through using a CHI 660E electrochemical workstation (Shanghai Chenhua Instrument Co., Ltd, Shanghai, China) in a three-electrode system with a 1.0 M KOH electrolyte at room temperature. The working electrode was Er_0.4_ Fe-MOF/NF, the counter electrode was a graphite plate, and Hg/HgO was used as the reference electrode. All the potentials were reported as one form of the reversible hydrogen electrode (RHE), calculated using the Nernst equation: E (RHE) = E (Hg/HgO) + 0.059 × pH) V.

### 2.5. Characterizations

XRD data were acquired from a LabX XRD-6100 X-ray diffractometer with Cu Kα radiation (40 kV, 30 mA) and a 0.154 nm wavelength (Shimadzu, Osaka, Japan). SEM images were obtained using a XL30 ESEM FEG at a 20 kV accelerating voltage. The TEM and EDX data were collected using a FEI Tecnai G2 F20 (FEI Company, Hillsboro, OR, USA) and OXFORD X-max 80T (FEI Company, Hillsboro, OR, USA). A Thermo Scientific K-Alpha X-ray photoelectron spectrometer (Thermo Fisher Scientific, Waltham, MA, USA) using Al was used to acquire XPS spectra. Raman measurements were conducted on a Renishaw Invia spectrometer (Renishaw Company, Gloucestershire, England). Fourier-transform infrared (FTIR) spectroscopy was carried out on an FTIR spectrometer (Theromo Nicolet Corporation, Madison, WI, USA) using the potassium bromide pellet method at an ambient temperature.

## 3. Results

### 3.1. The Structure and Composition of Er_0.4_ Fe-MOF/NF

**Figure 1:**
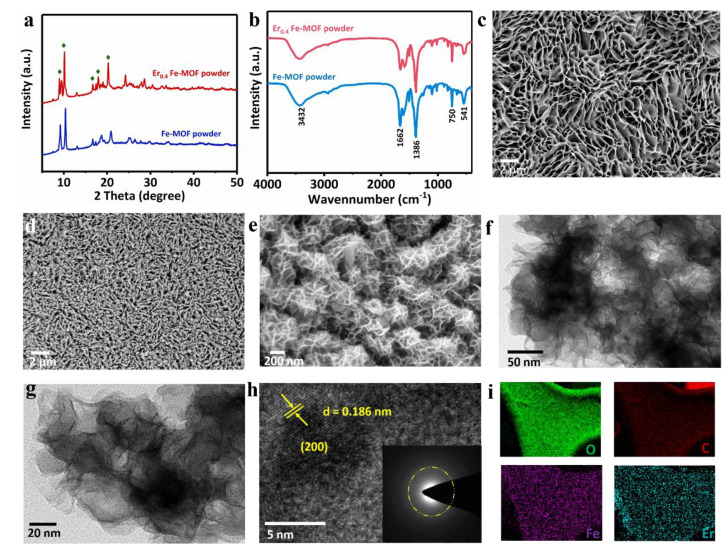
XRD patterns (**a**) and FTIR spectra (**b**) of Fe-MOF/NF and Er_0__.4_ Fe-MOF/NF. SEM image of Fe-MOF/NF (**c**). SEM images (**d**,**e**), TEM images (**f**,**g**), HRTEM image (**h**) (inset was the corresponding SAED pattern), and element mapping (**i**) of Er_0__.4_ Fe-MOF/NF.

The structure of Er_0.4_ Fe-MOF and Fe-MOF were characterized via X-ray diffraction (XRD), as shown in Figure 1a and Figure S1. The diffraction peaks at 8.9°, 11.4°, 15.6°, 18.7° and 20.8° (marked as “⧫”) were indexed to the typical MIL-53 structure [25]. Meanwhile, we prepared a series of catalysts with different Er/Fe molar ratios by adjusting the Er doping molar amount. As evidenced by Figure S1, the XRD patterns of these sample were similar, which excluded the formation of new crystal phases after Er doping. But the crystallinity of these catalysts increased with an increase in Er ions. Subsequently, the Fourier-transform infrared spectrum of Er_0.4_ Fe-MOF exhibited similar functional groups as those of Fe-MOF in the range of 4000–400 cm^−1^ (Figure 1b). The absorption bands at 750 cm^−1^ and 3432 cm^−1^ indicated the C–H bending vibration of the benzene ring and the O–H stretching vibration. The two peaks at 1662 and 1386 cm^−1^ contributed to the symmetric vibration of carboxyl groups. The peak at 541 cm^−1^ was observed to be metal-oxygen bond (Fe–O) vibrations, further demonstrating the coordination structure of MOFs [26].

Raman spectra, providing information about chemical bonding, displayed two peaks at 1613 and 1442 cm^−1^, attributed to the stretching modes of the carboxylate group (Figure S2) [27,28]. Moreover, the out-of-plane deformation modes of the C–H groups appeared at 866, 634 cm^−1^ [28]. In addition, the as-prepared Er_0.4_ Fe-MOF was uniformly grown on the surface of the 3D nickel foam in the form of a nanoarray structure, which was much thinner than the Fe-MOF (Figure 1c–e). The above structure could expose more specific surface area be beneficial to the mass transport for expediting the OER reaction. As shown in Figure 1f,g, ultrathin nanosheets loosely stacked could be easily observed using a transmission electron microscope (TEM). The high-resolution TEM image (HRTEM) in Figure 1h shows that the lattice distance with an interplanar spacing of 0.186 nm denoted the (200) planes of MIL-53, corresponding to selected-area electron diffraction (SAED) pattern [29,30]. The energy-dispersive X-ray spectroscopy (EDX) and mappings (Figure 1i) certified the existence of Er after Er doping and distribution over the entire catalyst (Figures S3 and S4). These test results demonstrated that Er ions were doped into the Fe-MOF host without the formation of an additional phase.

**Figure 2:**
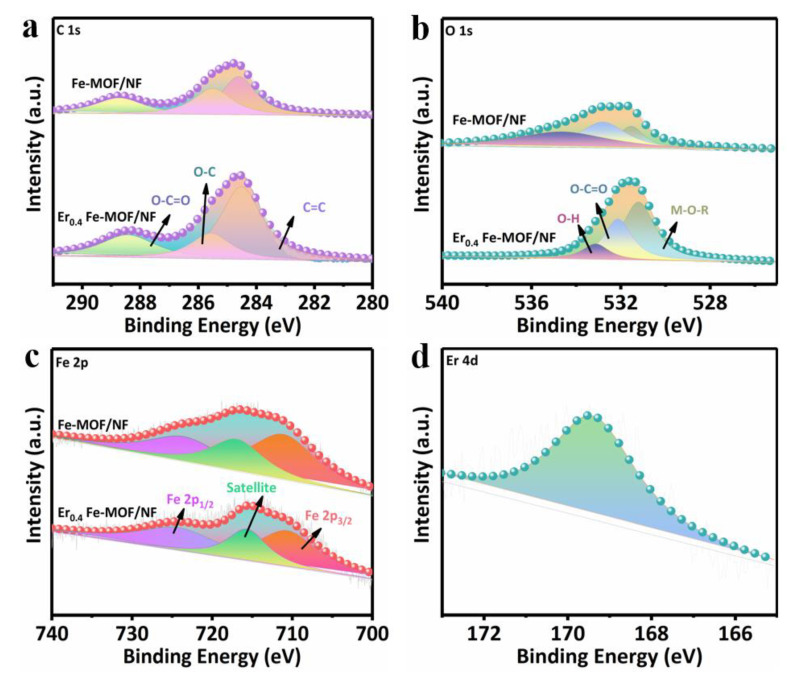
The XPS spectra of C 1s (**a**), O 1s (**b**), Fe 2p (**c**) and Er 4d (**d**) of Er_0.4_ Fe-MOF/NF and Fe-MOF/NF.

The chemical compositions of Er_0.4_ Fe-MOF/NF and Fe-MOF/NF were further characterized via X-ray photoelectron spectroscopy (XPS). The atomic percentages of O, Er, C, and Fe in the Er_0.4_ Fe-MOF surface were 54.34, 0.19, 43.57, and 1.89, respectively, which approximately agreed with the EDX results. For C 1s (Figure 2a), three typical peaks located at 284.53, 285.64, and 288.43 eV, corresponding to the benzene ring, C–O from the benzoate anions, and the carboxylate groups (O–C=O) in terephthalic acid [31], respectively. The binding energy of O 1s at 531.22, 532.15, and 533.12 eV could be assigned to oxygen atoms of the Fe–O bonds, the carboxylate groups of terephthalic acid, and absorbed water (Figure 2b) [31]. The C 1s and O 1s spectra of Er_0.4_ Fe-MOF/NF and Fe-MOF/NF had the same shape and binding energy, implying their identical chemical composition. Figure 2c shows the XPS spectra of Fe 2p with the peak position at around 710.88 eV and 724.13 eV, suggesting the valence state of Fe exists as Fe^3+^ in Er_0.4_ Fe-MOF/NF and Fe-MOF/NF [32]. In contrast, the binding energy of Fe 2p in Er_0.4_ Fe-MOF/NF showed a negative shift at about 0.5–0.95 eV, indicating the change redistribution occurs after Er doping [33]. The reason for this may be the different electronegativities of Fe (1.83) and Er (1.24), causing additional electron density donation from Er to Fe [34]. Er doping could change the electronic environment of Er_0.4_ Fe-MOF/NF, resulting in different adsorption energies of intermediates, and thus accelerating the whole reaction process [35,36]. For the Er 4d spectrum (Figure 2d), the peak at 169.52 eV confirms the Er element, further indicating the successful incorporation of Er into Fe-MOF/NF [37]. Compared with the single Er element (167.3 eV), the Er element in Er_0.4_ Fe-MOF/NF shifted to a positive binding energy, further demonstrating that the electrons may transfer from Er to Fe. Thus, deducing from all the above facts, the combination of Er and Fe could introduce surprising synergistic effects that may affect certain mechanisms through the modification of the electronic structures of host materials, leading to the promotion of the water splitting reaction.

### 3.2. Electrochemical Properties of Er_0.4_ Fe-MOF/NF Electrode Active Materials

**Figure 3:**
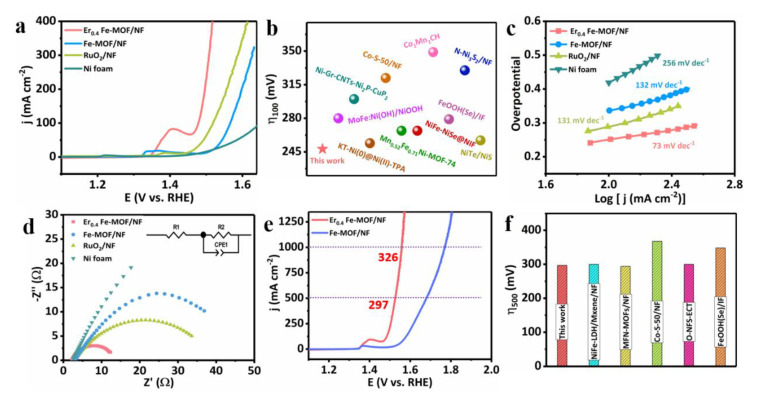
LSV curves of NF-based electrodes tested in 1.0 M KOH (**a**). Comparing the overpotential of Er_0.4_ Fe-MOF/NF at 100 mA cm^−2^ with other literatures (**b**). Tafel slopes (**c**) and EIS (**d**) of Ni foam, Fe-MOF/NF, commercial RuO_2_ and Er_0.4_ Fe-MOF/NF. LSV curves (**e**) and OER performance comparison histogram (**f**).

The electrocatalytic performance of the electrodes toward OER were tested in 1.0 M KOH solution through a typical three-electrode system at a scan rate of 2 mV s^−1^. All the overpotentials were given with iR-compensation unless specified [38]. Figure 3a showed the linear scan voltammetry (LSV) curves of bare NF, commercial RuO_2_/NF, precursor Fe-MOF/NF, and Er_0.4_ Fe-MOF/NF electrodes for comparison. A pronounced oxidation peak existed at around 1.41 V vs. RHE, which was attributed to the oxidation of Ni^2+^ to Ni^3+^ of the substrate NF [39]. The current density then increased monotonically with the increase of applied potential after the above oxidation peak. At 100 mA cm^−2^, the Er_0.4_ Fe-MOF/NF exhibited the lowest overpotential (248 mV), compared with bare NF (418 mV), precursor Fe-MOF/NF (336 mV) and commercial RuO_2_/NF (288 mV). It was notable that Er_0.4_ Fe-MOF/NF exhibited excellent electrocatalytic activity, surpassing most of the previous OER catalysts [40,41,42,43,44,45,46,47,48,49], as shown in Figure 3b and Table S3. Er_0.4_ Fe-MOF/NF needed an overpotential of 210 mV at low current density (10 mA cm^−2^) by the back sweep test (Figure S5). To get additional insights into the influence of the Er content on the OER activity, a series of Er-doped Fe-MOF/NF were prepared with varying ratios of Er and Fe. For a more intuitive representation, histograms of samples with different doping concentrations were drawn according to the overpotential of LSV polarization curves, as shown in Figure S6. Interestingly, with the increasing Er doping amount, the Er-doped Fe-MOF/NF needed lower overpotentials at current densities of 100 mA cm^−2^. But the activity decreased as the Er content further grew to 0.4 mmol or larger. The OER activity was positively linked to the Er doping content, highlighting the fact that the proper content of Er doping into MOFs could optimize OER activity. As displayed in Figure 3c, the Tafel slope of Er_0.4_ Fe-MOF/NF was determined to be 73 mV dec^−1^, which was much lower than those of Fe-MOF/NF (132 mV dec^−1^), commercial RuO_2_/NF (131 mV dec^−1^) and bare NF (256 mV dec^−1^), indicating the fast OER kinetics of Er_0.4_ Fe-MOF/NF. Excitingly, the low overpotential and Tafel slope of Er_0.4_ Fe-MOF/NF corroborated that the synergistic effects of Er and Fe ions provided a crucial factor the excellent alkaline OER catalytic activity. Furthermore, the electrochemical impedance spectroscopies (EISs) shown in Figure 3d revealed that Er_0.4_ Fe-MOF/NF had a smaller semicircle radius than those of Fe-MOF/NF, RuO_2_/NF, and NF, implying prominently improved interfacial electron-transfer kinetics over Er_0.4_ Fe-MOF/NF. The improved conductivity of Er_0.4_ Fe-MOF/NF revealed that the electronic structure of the sample had changed after Er doping, which agreed with the XPS results. Meanwhile, the EISs of Er-doped Fe-MOF/NF with different Er/Fe atomic ratios are displayed in Figure S7. Importantly, Er_0.4_ Fe-MOF/NF exhibited excellent OER catalytic performance at high current densities (including 500 mA cm^−2^ and 1000 mA cm^−2^). As shown in Figure 3e, Er_0.4_ Fe-MOF/NF achieved overpotentials of 297 and 326 mV to drive 500 and 1000 mA cm^−2^ current densities, respectively, lower than that of Fe-MOF/NF (η_500_ = 448 mV; η_1000_ = 539 mV), to meet the strict criteria of industrial applications. As revealed in Figure 3f and Table S4, Er_0.4_ Fe-MOF/NF was comparable to the other reported state-of-the-art catalysts (Figure 3f and Table S4) [43,49,50,51,52].

**Figure 4:**
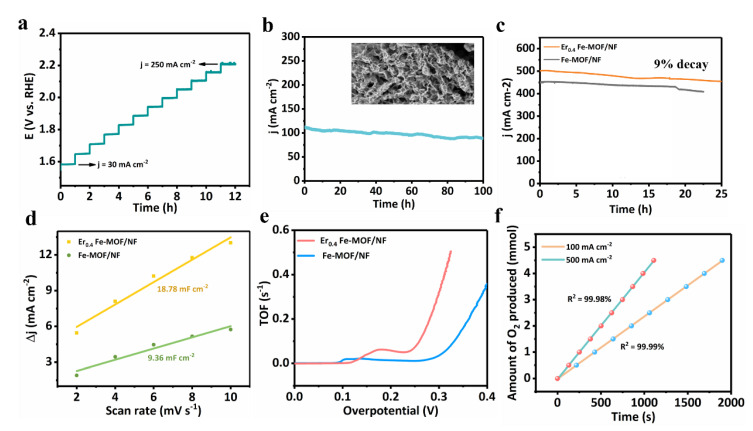
The multi-current process curve of Er_0.4_ Fe-MOF/NF (**a**) obtained at current densities of 30–250 mA cm^−2^, every 3600 s, respectively. Chronoamperometric (i-t) curves at current densities of 100 mA cm^−2^ (**b**), inset was the SEM image after testing. Chronoamperometric curves of Er_0.4_ Fe-MOF/NF and Fe-MOF/NF (**c**). ECSA evolutions (**d**) and TOF values (**e**) of Er_0.4_ Fe-MOF/NF and Fe-MOF/NF. Experimental amounts of O_2_ generated by the Er_0.4_ Fe-MOF/NF electrode at 100 and 500 mA cm^−2^ (**f**).

Figure 4a displayed a continuous multistep chronopotentiometry curve for Er_0.4_ Fe-MOF/NF. The initial current density was 30 mA cm^−2^ and the corresponding potential stabilized at the initial current density, remaining unchanged for 3600 seconds. Similar results have been observed for current values (30–250 mA cm^−2^), indicating excellent mass transfer, electric conductivity, and mechanical robustness of Er_0.4_ Fe-MOF/NF [53]. The OER stability of MOFs at large current densities is very important for industrial applications. Obviously, the OER performance of Er_0.4_ Fe-MOF/NF could be well maintained for at least 100 h at the large current density of 100 mA cm^−2^ without obvious degradation. The SEM image (the insert in Figure 4b) and XPS spectra (Figure S8) of Er_0.4_ Fe-MOF/NF display its original morphology and composition after long-term OER testing, suggesting excellent durability of the Er_0.4_ Fe-MOF/NF catalyst. However, the peaks of O 1s originated from O–H (533.12 eV), the carboxyl group (532.15 eV), and M–O (531.22 eV) shifted to the lower binding energy of 532/530.7 eV, demonstrating the possible formation of oxyhydroxide during OERs [26]. Furthermore, for commercial applications, the stability of electrocatalysis under large current densities was essential for large-scale production because the intrinsic instability of MOFs and the number of gas bubbles generated. As a result, it was evident that just 9% decay in current densities could be observed for the 500 mA cm^−2^ case after 25 h, while the Fe-MOF/NF catalyst dropped quickly under the same test conditions, as shown in Figure 4c. Subsequently, as shown in Figure S9, the obtained LSV curves after the 1st and the 1000th cyclic CV sweeps exhibited seldom decay. These test results further confirmed the positive effect of Er doping in enhancing both the OER activity and stability of Fe-MOF/NF.

The mechanism of OER could be assigned to the existence of the two pairs of redox peaks in Figure S10. In the forward half cycle, the oxidation peak can be attributed to the oxidation of Fe ^3+^ to Fe ^4+^ at about 1.54 V vs. RHE. Moreover, in the reverse half-cycle, the peak located at 1.15 V vs. RHE can be attributed to the reduction of Fe ^4+^ to Fe ^3+^ [54]. In contrast, the oxidation peak and reduction peak of bare NF were almost negligible in the same voltage range, indicating the promotion of OERs came from Er_0.4_ Fe-MOF instead of the substrate NF. Obviously, the intensity redox peaks of Er_0.4_ Fe-MOF/NF were higher than those of the precursor Fe-MOF/NF due to the synergistic effect between Er and Fe sites. Based on the above test results, the OER performance of Er_0.4_ Fe-MOF/NF could be assigned to the following steps: [54]

A + OH^−^ ↔ AOH + e^−^ (1)

AOH + OH^−^ ↔ AO + H_2_O + e^−^ (2)

AO + OH^−^ ↔ AOOH + e^−^ (3)

AOOH + OH^−^ ↔ A+ O_2_ +H_2_O + e^−^ (4)

where A denotes active sites of Er_0.4_ Fe-MOF/NF. Thus, Fe sites served as the active sites in this electrode.

The double-layer capacitances (C_dl_) obtained from CV curves (Figure S11) were calculated to evaluate the electrochemically active surface area [55]. In Figure 4d, the C_dl_ value of Er_0.4_ Fe-MOF/NF was calculated to be 18.78 mF cm^−2^, significantly higher than that of the precursor Fe-MOF/NF (9.36 mF cm^−2^). The reason was attributed to the high exposure of active sites provided by the ultrathin nanoarray structure. Similarly, the intrinsic catalytic performance of this electrode was also evaluated by the turnover frequency (TOF). The calculated TOF values were tested through CV curves (Figure S12) based on a previous report. [56] As shown in Figure 4e, Er_0.4_ Fe-MOF/NF achieved a TOF value of 0.3 O_2_ s^−1^ while that of Fe-MOF/NF was only 0.04 O_2_ s^−1^ at the same overpotential of 300 mV. The faradic efficiency (FE) for OER was calculated by comparing the amount of estimated oxygen gas volume using both the experimental and theoretical methods under 100 mA cm^−2^ and 500 mA cm^−2^ current densities of Er_0.4_ Fe-MOF/NF (assuming 100% FE), as shown in Figure 4f. Virtually 100% FE was obtained, indicating most of the surface electrons participated the OER process and the charge transfer process exhibited a minimum blocking resistance [57].

## 4. Conclusions

In summary, with optimized Er doping, ultrahigh activity and stability for OER electrocatalysts has been achieved using a one-step solvothermal method. Compared with the precursor Fe-MOF/NF, the Er_0.4_ Fe-MOF/NF electrode showed OER performance can yield a 100 mA cm^−2^ current density at an overpotential of 248 mV with long-term electrochemical durability for at least 100 h. Moreover, it also exhibited ultralow overpotentials of 297 and 326 mV to reach the large current densities of 500 and 1000 mA cm^−2^. Overall, the addition of Er dopants could have a significant impact on OER catalytic performance due to the optimized electronic structure of the active center. 

## Data Availability

The data presented in this study are available on request from the corresponding author.

## Supplementary Materials

The following are available online at https://www.mdpi.com/article/10.3390/nano11071847/s1, Figure S1: The XRD patterns of different Er content of Er-doped Fe-MOF/NF, Figure S2: Raman spectra of Er_0.4_ Fe-MOF/NF and Fe-MOF/NF, Figure S3: The EDX spectrum of Er_0.4_ Fe-MOF/NF, Table S1: The element percentages Wt% of Er_0.4_ Fe−MOF/NF, Figure S4: The EDX spectrum of Fe-MOF/NF, Table S2: The element percentages Wt% of Fe−MOF/NF, Figure S5: LSV of Er_0.4_ Fe-MOF/NF for OER in 1.0 M KOH, Figure S6: LSV curves (a) and comparing the overpotential of different Er content of Er-doped Fe-MOF/NF at 100 mA cm^−2^ (b,c), Figure S7: EIS of different Er content of Er-doped Fe-MOF/NF, Figure S8: The XPS spectra of C 1s (a), O 1s (b), Fe 2p (c) and Er (d) after i-t testing, Figure S9: LSV curves of Er_0.4_ Fe-MOF/NF before and after 1000 CV cycles in 1.0 M KOH, Figure S10: CV curves of Er_0.4_ Fe-MOF/NF (a) and Fe-MOF/NF (b) (inset image was the CV of bare NF) at a scan rate of 5 mV s^−1^, Figure S11: ECSA evaluations of Er_0.4_ Fe-MOF/NF (a,b) and Fe-MOF/NF (c,d), Figure S12: TOF evaluations of Er_0.4_ Fe-MOF/NF (a,b) and Fe-MOF/NF (c,d) with various scan rates (20, 40, 60, 80,100 mV s^−1^), Table S3: Comparison of catalytic performance of Er_0.4_ Fe-MOF/NF with other reported OER catalysts, Table S4: Comparison of catalytic performance of Er_0.4_ Fe-MOF/NF with other reported OER catalysts at large current densities (above 250 mA cm^−2^).
