# Supplementary material for: Highly Enhanced OER Performance by Er-Doped Fe-MOF Nanoarray at Large Current Densities
Source: Nanomaterials (Basel). 2021 Jul 16;11(7):1847. doi: 10.3390/nano11071847 (PMC8308314; doi:10.3390/nano11071847)
Supplement: Supplementary file 1 [file nanomaterials-11-01847-s001.zip › nanomaterials-1292960-supplementary.pdf]

nanomaterials

Supplementary Materials

Highly Enhanced OER Performance by Er-Doped Fe-MOF Nanoarray at Large Current Densities

Yan Ma [1], Yujie Miao [1], Guomei Mu [1], Dunmin Lin [1], Chenggang Xu [1], Wen Zeng [2] and Fengyu Xie *[1]

[1] College of Chemistry and Materials Science, Sichuan Normal University, No. 5, Jing'an Road, Chengdu 610068, China; 20191201038@stu.sicnu.edu.cn (Y.M.); yujie@stu.sicnu.edu.cn (Y.M.); guomei@stu.sicnu.edu.cn (G.M.); ddmd222@sicnu.edu.cn (D.L.); chenggangxu@163.com (C.X.)
[2] School of Chemistry and Chemical Engineering, Chongqing University, Shapingba District, Chongqing 401331, China; wenzeng@cqu.edu.cn
* Correspondence: xiefengyu@sicnu.edu.cn

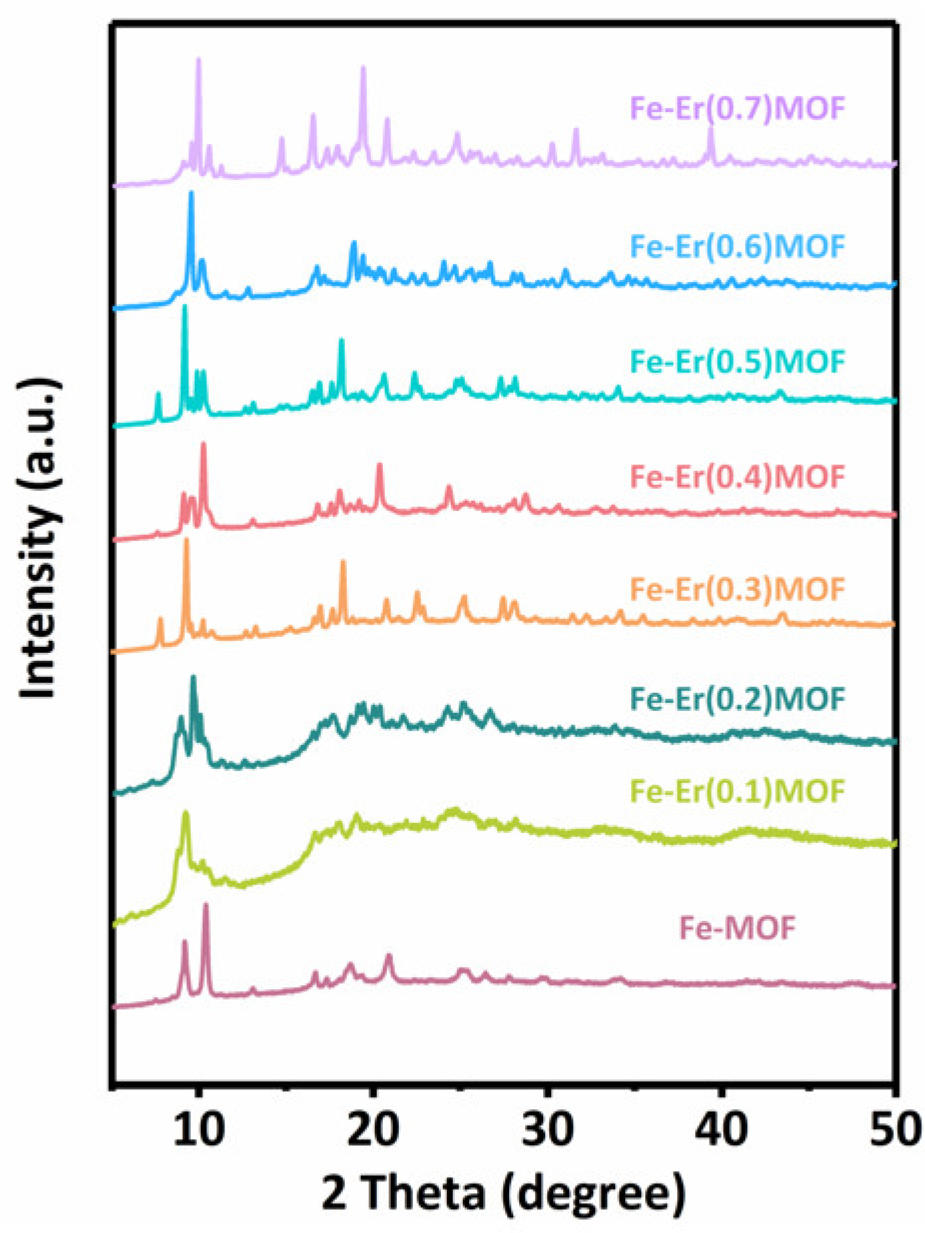

Figure S1. The XRD patterns of different Er contents of Er-doped Fe-MOF/NF.

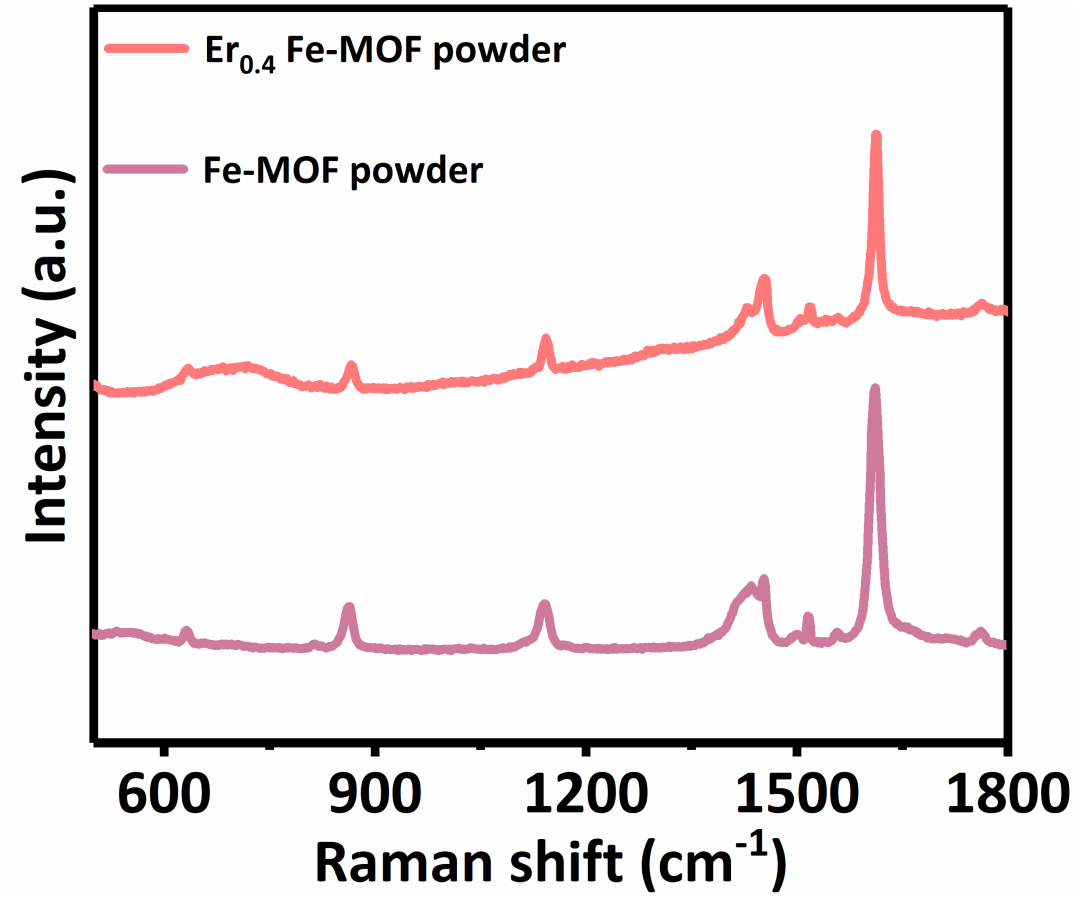

Figure S2. Raman spectra of $Er_{0.4}$ Fe-MOF/NF and Fe-MOF/NF.

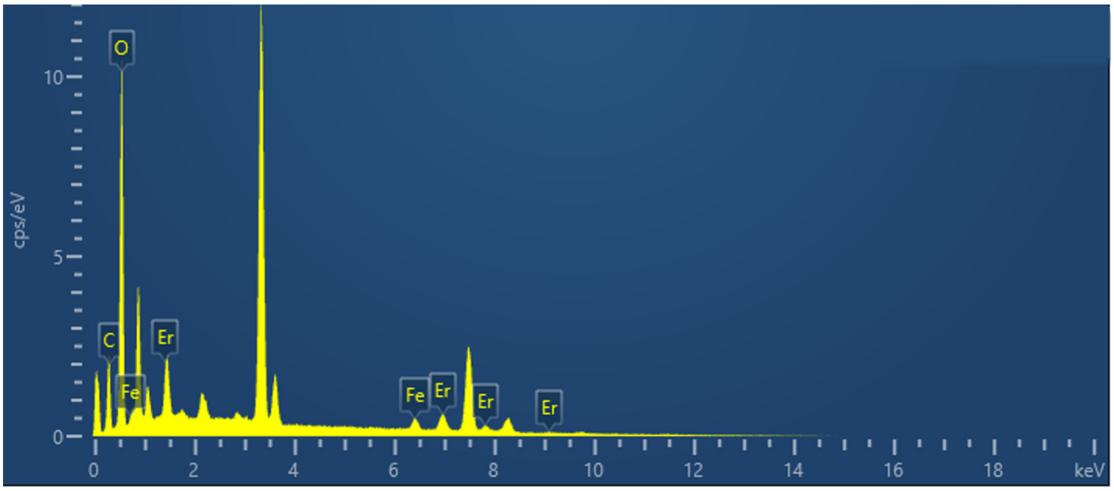

Figure S3. The EDX spectrum of $Er_{0.4}$ Fe-MOF/NF.

Table S1. The element percentages Wt% of $Er_{0.4}$ Fe-MOF/NF.

Element	Line Type	Atomic Percentage	Wt%	Wt% Sigma
**O**	K series	57.22	43.29	0.45
**Er**	L series	3.86	30.52	0.61
**C**	K series	36.94	20.98	0.38
**Fe**	K series	1.97	5.21	0.23
**Total:**		100.00	100.00	

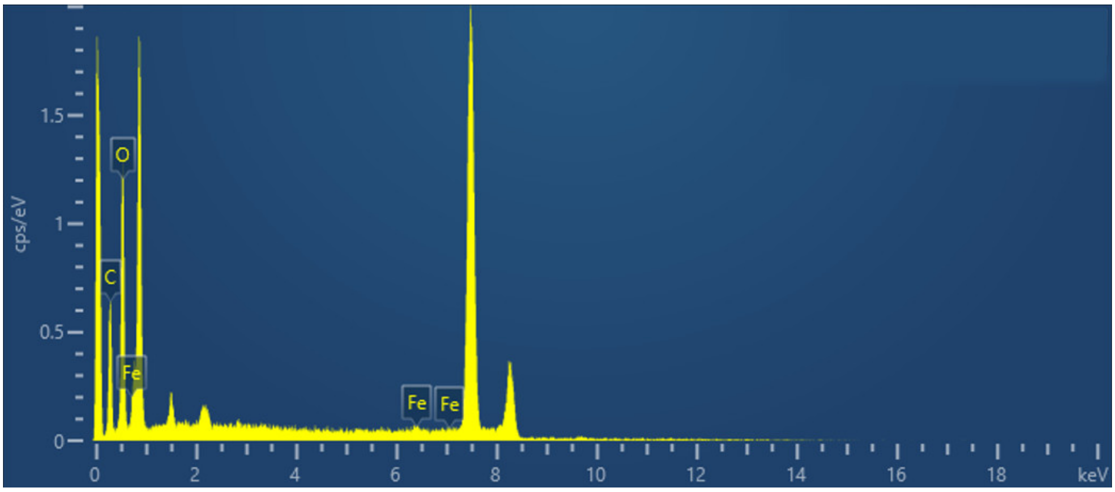

Figure S4. The EDX spectrum of Fe-MOF/NF.

Table S2. The element percentages Wt% of Fe-MOF/NF.

Element	Line Type	Atomic Percentage	Wt%	Wt% Sigma
O	K series	50.93	56.72	1.07
C	K series	48.34	40.41	1.00
Fe	K series	0.74	2.87	0.95
Total:		100.00	100.00	

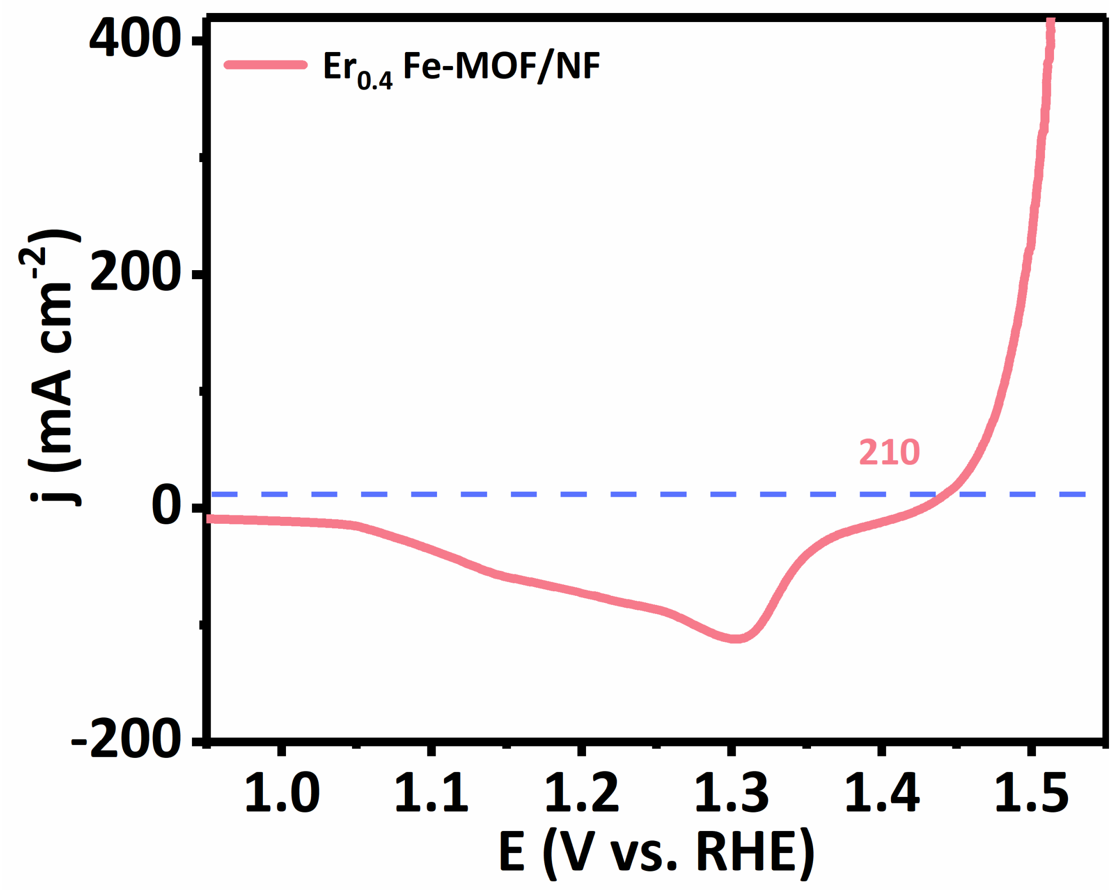

Figure S5. LSV curve of Er$_{0.4}$ Fe-MOF/NF for OER in 1.0 M KOH.

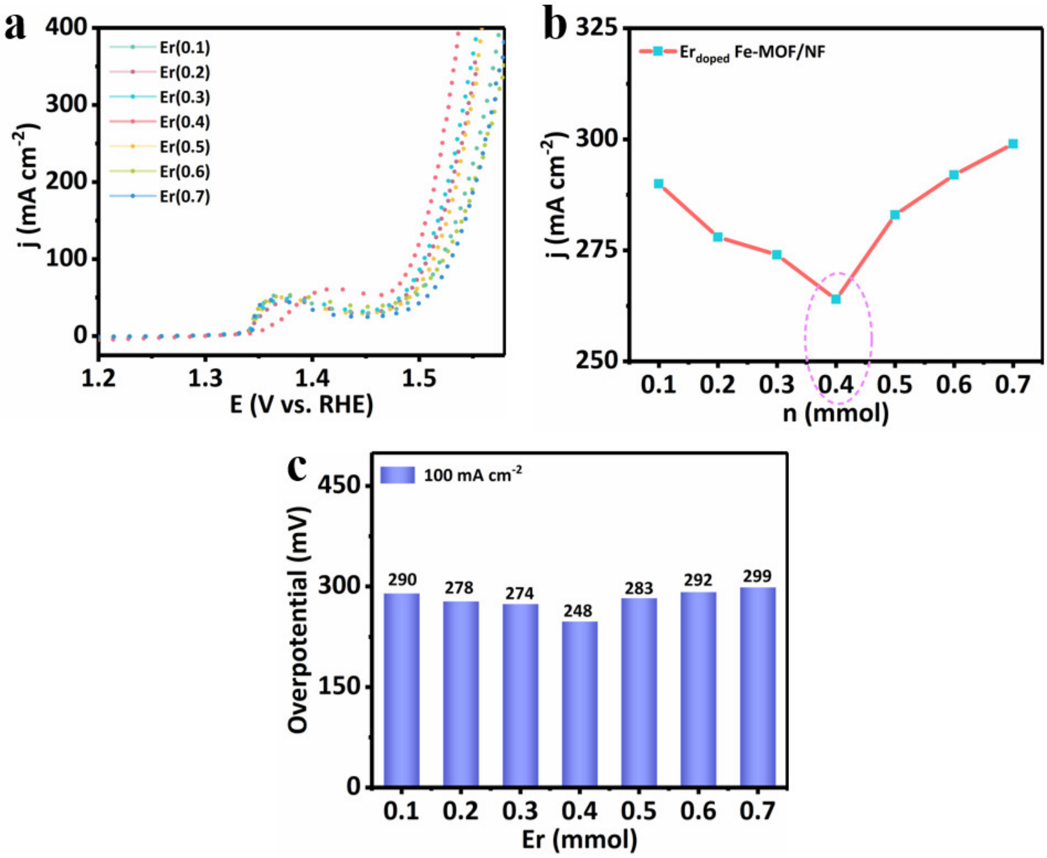

Figure S6. LSV curves (**a**) and comparison of the overpotential of different Er contents of Er-doped Fe-MOF/NF at 100 mA cm^{-2} (**b**,**c**).

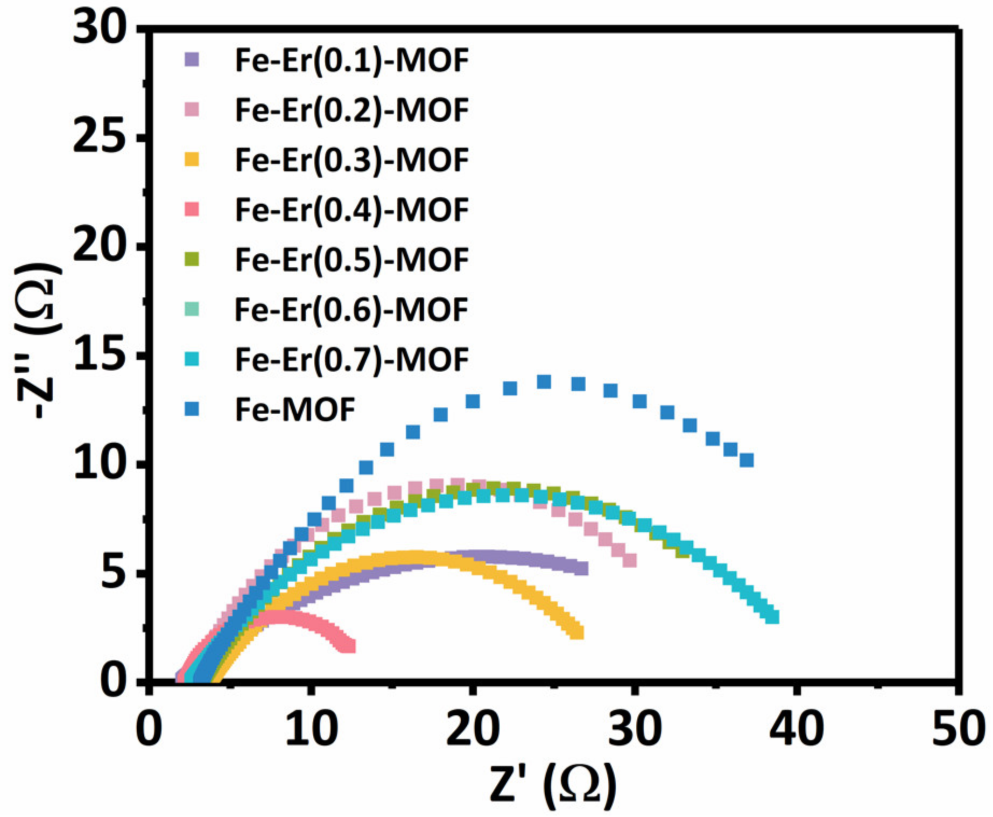

Figure S7. EIS of different Er contents of Er-doped Fe-MOF/NF.

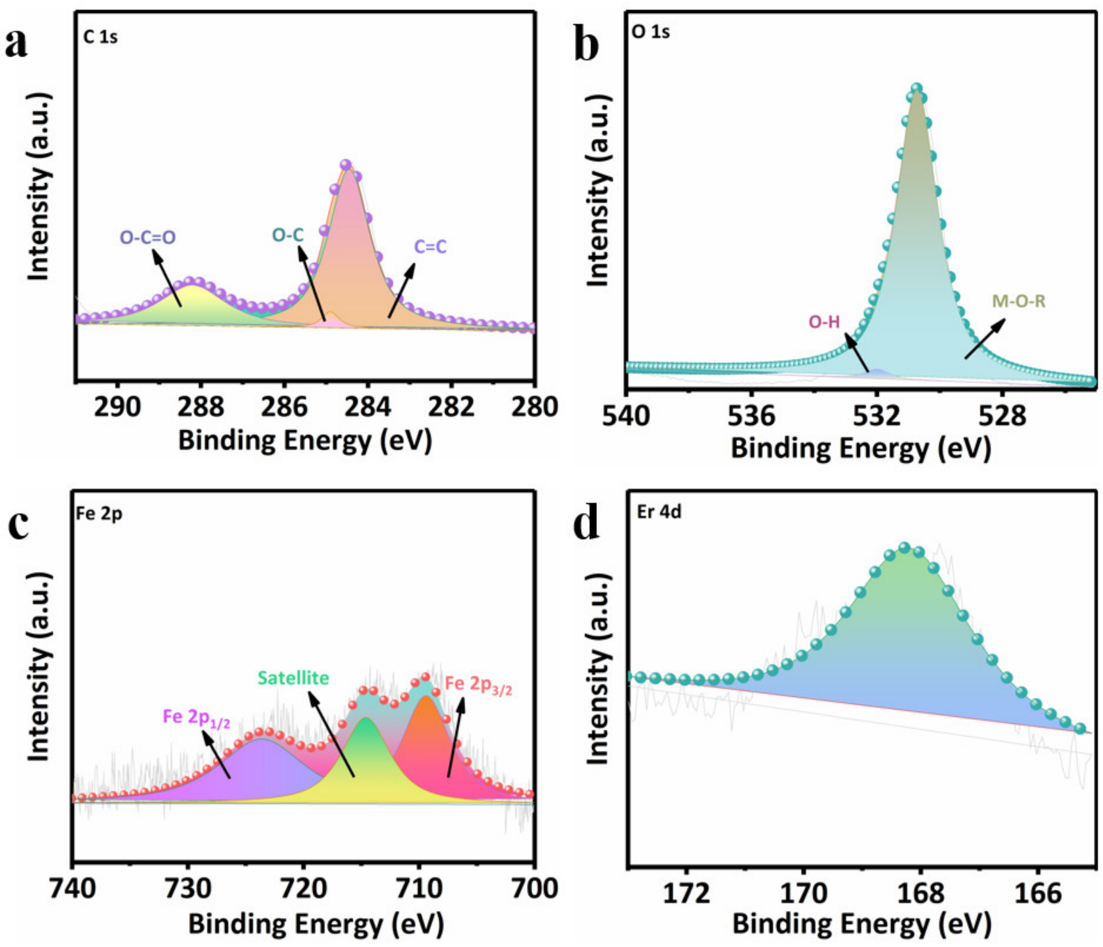

Figure S8. The XPS spectra of C 1s (**a**), O 1s (**b**), Fe 2p (**c**), and Er 4d (**d**) after i-t testing.

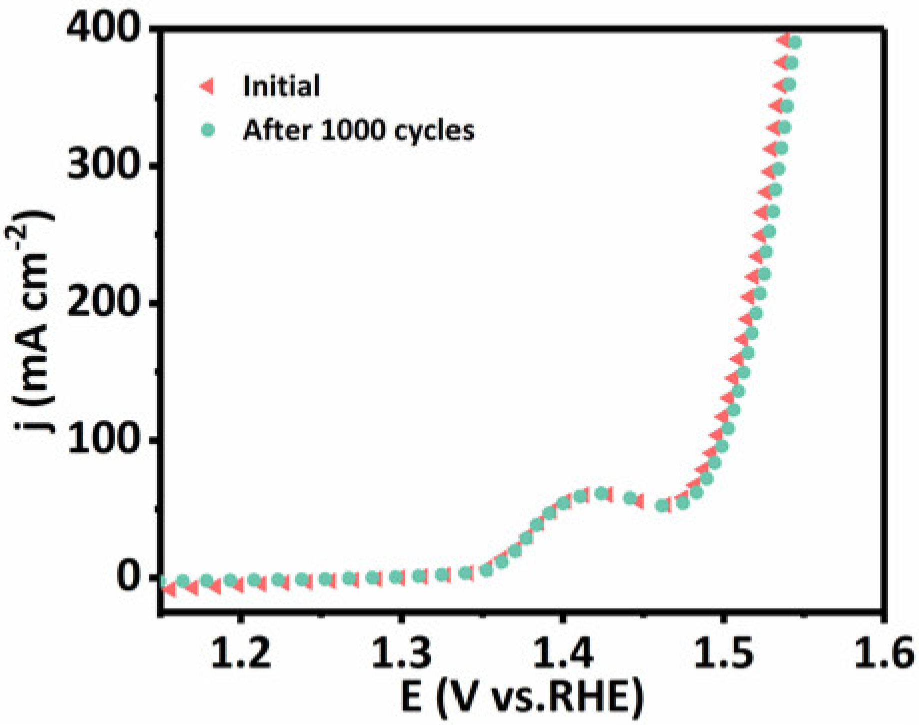

Figure S9. LSV curves of $Er_{0.4}$ Fe-MOF/NF before and after 1000 CV cycles in 1.0 M KOH.

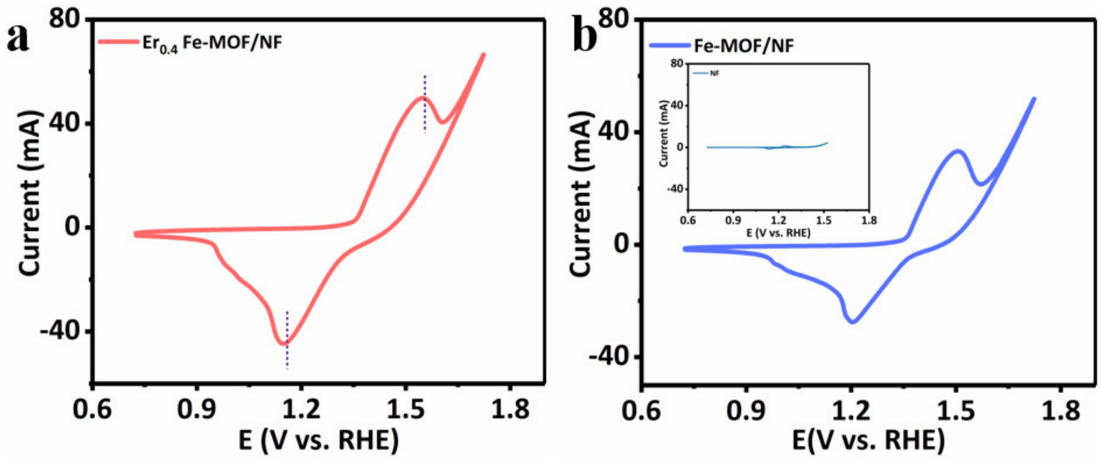

Figure S10. CV curves of $Er_{0.4}$ Fe-MOF/NF (**a**) and Fe-MOF/NF (**b**) (inset image is the CV of bare NF) at a scan rate of 5 mV s^{-1}.

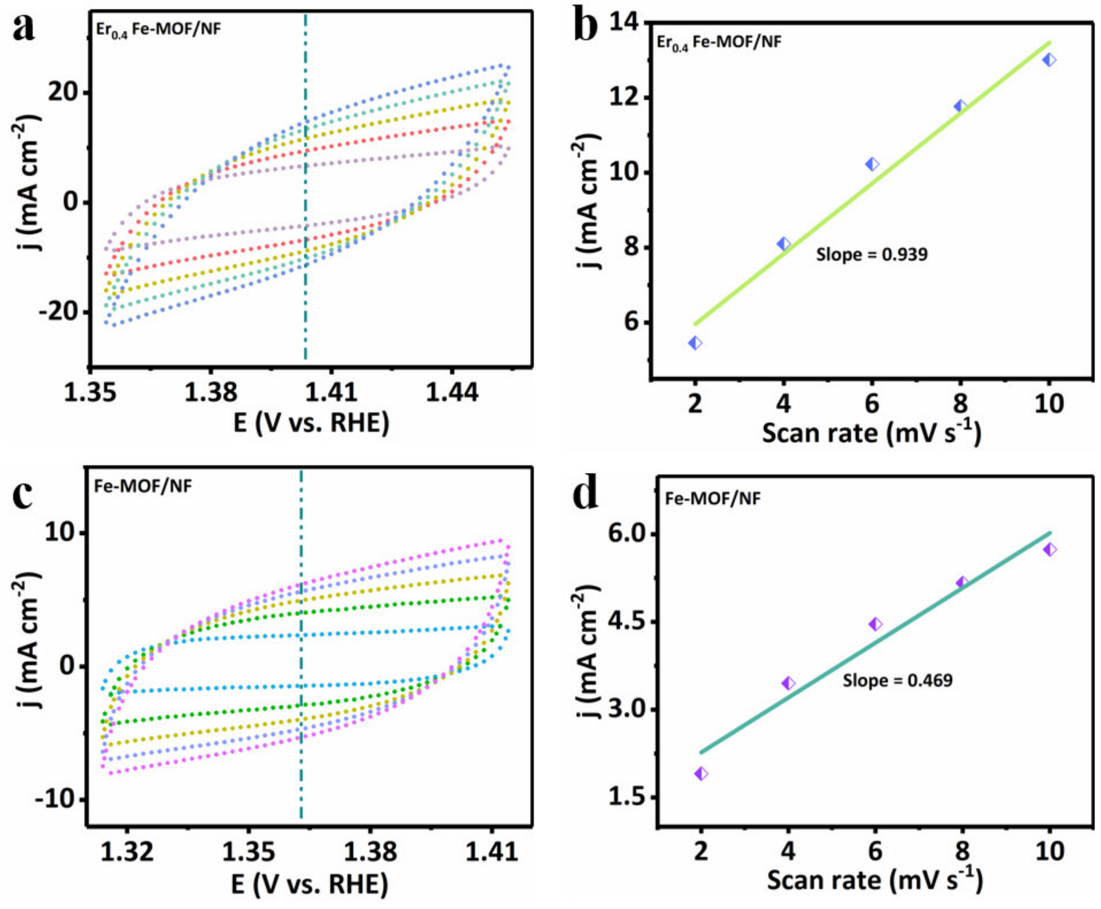

Figure S11. ECSA evaluations of $Er_{0.4}$ Fe-MOF/NF (**a-b**) and Fe-MOF/NF (**c-d**).

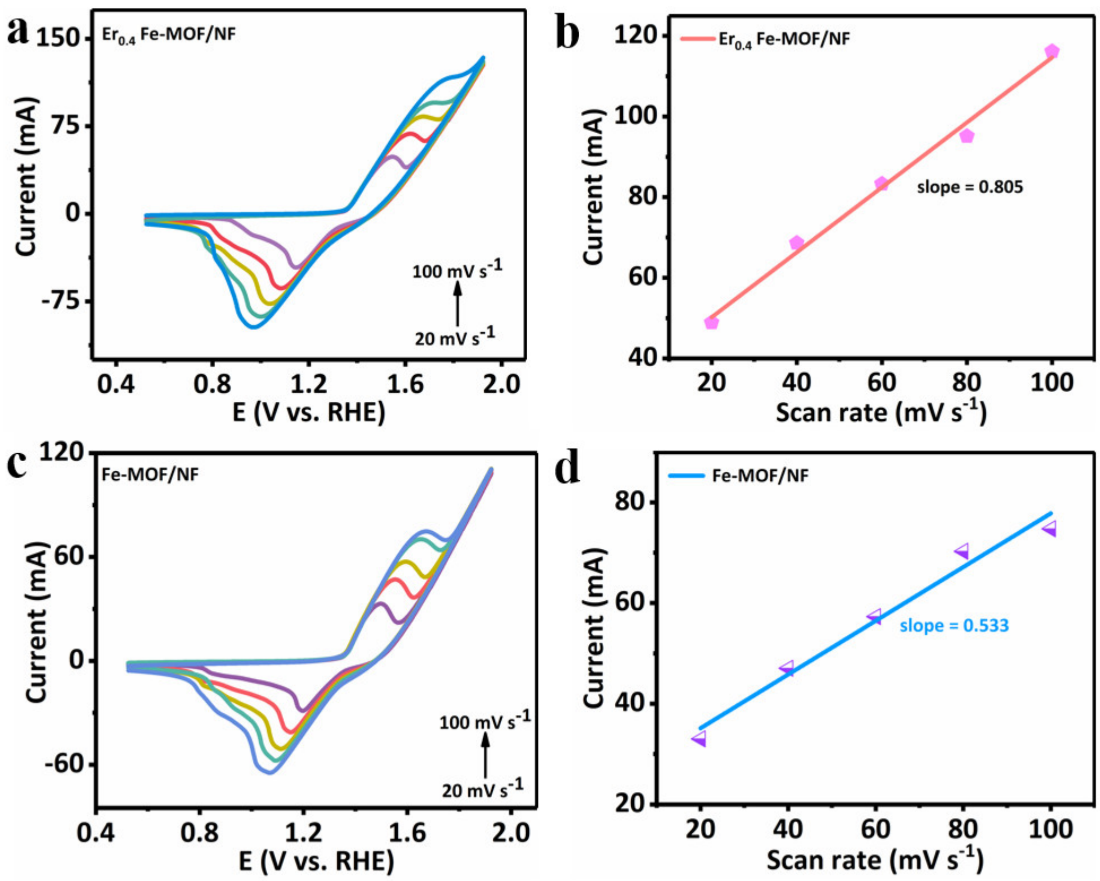

Figure S12. TOF evaluations of $Er_{0.4}$ Fe-MOF/NF (**a-b**) and Fe-MOF/NF (**c-d**) with various scan rates (20, 40, 60, 80,100 mV s^{-1}).

Table S3. Catalytic performance comparison of $Er_{0.4}$ Fe-MOF/NF against other reported OER catalysts.

Catalyst	Current Density (mA cm^{-2})	Overpotential (mV)	Electrolyte	Ref.
$Er_{0.4}$ Fe-MOF/NF	100	248	1.0 M KOH	This work
NiTe/NiS	100	257	1.0 M KOH	[1]
N-Ni_3S_2/NF	100	330	1.0 M KOH	[2]
FeOOH(Se)/IF	100	279	1.0 M KOH	[3]
Co_1Mn_1CH/NF	30<br>50	294<br>322	1.0 M KOH	[4]
N,Fe-NiSe@NIF	100	267	1.0 M KOH	[5]
$Mn_{0.52}Fe_{0.71}$Ni-MOF-74	100	267	1.0 M KOH	[6]
Co-S-50/NF	100	322	1.0 M KOH	[7]
KT-Ni(0)@Ni(II)	100	254	1.0 M KOH	[8]
Ni-Gr-CNTs-Ni_2P-CuP_2	100	300	1.0 M KOH	[9]
MoFe:$Ni(OH)_2$/NiOOH	100	280	1.0 M KOH	[10]
Co-N_x/C NRA	10	300	1.0 M KOH	[11]
Fe-Ni_3S_2/FeNi	10	282	1.0 M KOH	[12]
Fe-$Ni(OH)_2$/NF	10	270	1.0 M KOH	[13]
Fe-CoOOH/G	10	330	1.0 M KOH	[14]
Ni_3S_2@NiV-LDH/NF	100	330	1.0 M KOH	[15]
P-NiCoV-LTH/NF	100	285	1.0 M KOH	[16]

Table S4. Catalytic performance comparison of $Er_{0.4}$ Fe-MOF/NF against other reported OER catalysts at large current densities (above 250 mA cm^{-2}).

Catalyst	Overpotential (mV) @ Current Density (mA cm^{-2})	Electrolyte	Ref.
$Er_{0.4}$ Fe-MOF/NF	297 @ 500<br>326 @ 1000	1.0 M KOH	This work
Co-S-50/NF	368 @ 500	1.0 M KOH	[7]
$Ni_{90}Fe_{10}$-PC3000-2A	371 @ 400	1.0 M KOH	[17]
Fe_2O_3@Ni_2P/$Ni(PO_3)_2$	340 @ 500<br>370 @ 1000	1.0 M KOH	[18]
$FeCoNiP_{0.5}S_{0.5}$/Ti foil	360 @ 1000	1.0 M KOH	[19]
Porous Co-P/CF	341 @ 500<br>380 @ 1000	1.0 M KOH	[20]
P-NiCoV-LTH/NF	340 @ 500<br>373 @ 1000	1.0 M KOH	[16]
(Ni-MoO_2)@C/NF	340 @ 500<br>365 @ 1000	1.0 M KOH	[21]

References

1. Xue, Z.; Li, X.; Liu, Q.; Cai, M.; Liu, K.; Liu, M.; et al. Interfacial electronic structure modulation of NiTe nanoarrays with NiS nanodots facilitates electrocatalytic oxygen evolution. *Adv. Mater.* **2019**, *31*, 1900430.
2. Chen, P.; Zhou, T.; Zhang, M.; Tong, Y.; Zhong, C.; Zhang, N.; et al. 3D nitrogen-anion-decorated nickel sulfides for highly efficient overall water splitting. *Adv. Mater.* **2017**, *29*, 1701584.
3. Niu S.; Jiang W.J.; Wei, Z.; Tang, T.; Ma, J.; Hu, J.S.; et al. Se-doping activates FeOOH for cost-effective and efficient electrochemical water oxidation. *J. Am. Chem. Sci.* **2019**, *17*, 7005-7013.
4. Tang, T.; Jiang, W.J.; Niu, S.; Liu, N.; Luo, H.; Chen, Y.Y.; et al. Electronic and morphological dual modulation of colbat carbonate hydroxides by Mn doping towards highly efficient and stable bifunctional electrocatalysts for overall water splitting. *J. Am. Chem. Sci.* **2017**, *24*, 8320-8328.
5. Chen, J.; Chen, J.; Cui, H.; Wang, C. Electronic structure and crystalline phase dual modulation via anion-cation Co-doping for boosting oxygen evolution with long-term stability under large current density. *ACS Appl. Mater. Inter.* **2019**, *11*, 34819-34826.
6. Zhou, W.; Xue, Z.; Liu, Q.; Li, Y.; Hu, J.; Li, G. Trimetallic MOF-74 films grown on Ni foam as bifunctional electrocatalysts for overall water splitting. *ChemSusChem* **2020**, *13*, 5647-5653.
7. Yang, W.; Zeng, J.; Hua, Y.; Xu, C.; Siwal, S.S.; Zhang, Q. Defect engineering of cobalt microspheres by S doping and electrochemical oxidation as efficient bifunctional and durable electrocatalysts for water splitting at high current densities. *J. Power Sources* **2019**, *436*, 226887.
8. Hu, Q.; Wang, Z.; Huang, X.; Qin, Y.; Yang, H.; Ren, X.; et al. Integrating well-controlled core-shell structures into "superaerophobic" electrodes for water oxidation at large current densities. *Appl. Catal. B: Environ.* **2021**, *286*, 119920.
9. Riyajuddin, S.; Azmi, K.; Pahuja, M.; Kumar, S.; Maruyama, T.; Bera, C.; et al. Super-hydrophilic hierarchical Ni-foam-graphene-carbon nanotubes-Ni_2P-CuP_2 nano-architecture as efficient electrocatalyst for overall water splitting. *ACS Nano* **2021**, *15*, 5586-5599.
10. Jin, Y.; Huang, S.; Yue, X.; Du, H.; Shen, P.K. Mo- and Fe-modified $Ni(OH)_2$/NiOOH nanosheets as highly active and stable electrocatalysts for oxygen evolution reaction. *ACS Catal.* **2018**, *8*, 2359-2363.
11. Amiinu, I.S.; Liu, X.; Pu, Z.; Li, W.; Li, Q.; Zhang, J.; et al. From 3D ZIF nanocrystals to Co-N_x/C nanorod array electrocatalysts for ORR, OER, and Zn-Air batteries. *Adv. Funct. Mater.* **2018**, *28*, 1704638.
12. Yuan, C.Z.; Sun, Z.T.; Jiang, Y.F.; Yang, Z.K.; Jiang, N.; Zhao, Z.W.; et al. One-step in situ growth of iron-nickel sulfide nanosheets on FeNi alloy foils: high-performance and self-supported electrodes for water oxidation. *Small* **2017**, *13*, 1604161.
13. Liu, J.; Zhu, D.; Zheng, Y.; Vasileff, A.; Qiao, S.Z. Self-supported earth-abundant nanoarrays as efficient and robust electrocatalysts for energy-related eeactions. *ACS Catal*. **2018**, *8*, 6707-6732.
14. Han, X.; Yu, C.; Zhou, S.; Zhao, C.; Huang, H.; Yang, J.; et al. Ultrasensitive iron-triggered nanosized Fe-CoOOH integrated with graphene for highly efficient oxygen evolution. *Adv. Energy Mater*. **2017**, *7*, 1602148.
15. Liu, Q.; Huang, J.; Zhao, Y.; Cao, L.; Li, K.; Zhang, N.; et al. Tuning the coupling interface of ultrathin Ni_3S_2@NiV-LDH heterogeneous nanosheet electrocatalysts for improved overall water splitting. *Nanoscale* **2019**, *11*, 8855-8863.
16. Liu, Q.; Huang, J.; Zhang, X.; Cao, L.; Yang, D.; Kim, J.; Feng, L. Controllable conversion from single-crystal nanorods to polycrystalline nanosheets of NiCoV-LTH for oxygen evolution reaction at large current density. *ACS Sustain. Chem. Eng.* **2020**, *43*, 16091-16096.

17. Huang, C.L.; Chuah, X.F.; Hsieh, C.T.; Lu, S.Y. NiFe alloy nanotube arrays as highly efficient bifunctional electrocatalysts for overall water splitting at high current densities. *ACS Appl. Mater. Inter.* **2019**, *11*, 24096-24106.
18. Cheng, X.; Pan, Z.; Lei, C.; Jin, Y.; Yang, B.; Li, Z.; et al. A strongly coupled 3D ternary Fe_2O_3@$Ni_2P/Ni(PO_3)_2$ hybrid for enhanced electrocatalytic oxygen evolution at ultra-high current densities. *J. Mater. Chem. A* **2019**, *7*, 965-971.
19. Wang, X.; Ma, W.; Ding, C.; Xu, Z.; Wang, H.; Zong, X.; Li, C. Amorphous multi-elements electrocatalysts with tunable bifunctionality towards overall water splitting. *ACS catal*. **2018**, *11*, 9926-9935.
20. Li, Y.; Wei, B.; Yu, Z.; Bondarchuk, O.; Araujo, A.; Amorim, I.; et al. Bifunctional porous cobalt phosphide foam for high-current density alkaline water electrolysis with 4000-hour long stability. *ACS Sustain. Chem. Eng.* **2020**, *8*, 10193-10200.
21. Qian, G.; Chen, J.; Luo, L.; Yu, T.; Wang, Y.; Jiang, W.; et al. Industrially promising nanowire heterostructure catalyst for enhancing overall water splitting at large current density. *ACS Sustain. Chem. Eng.* **2020**, *8*, 12063-12071.
